# Efficacy of vitamin C on chemotherapy-related anemia in pancreatic cancer: study protocol for a randomized controlled trial

**DOI:** 10.1186/s13063-024-08345-w

**Published:** 2024-07-29

**Authors:** Xinyue Wang, Xinzhe Zhu, Yi Liu, He Liu, Zhiwen Xiao, Guopei Luo

**Affiliations:** 1 Department of Pancreatic Surgery, Fudan University Shanghai Cancer Center, Xuhui District, No. 270, Dong’An Road, Shanghai, 200032 China; 2 Department of Oncology, Shanghai Medical College, Fudan University, Shanghai, China; 3 Shanghai Pancreatic Cancer Institute, Shanghai, China; 4 Pancreatic Cancer Institute, Fudan University, Shanghai, China

**Keywords:** Pancreatic cancer, Vitamin C, Anemia, Chemotherapy

## Abstract

**Background:**

In the treatment of advanced pancreatic cancer, chemotherapy plays a pivotal role. Despite its effectiveness, this regimen is often marred by side effects such as anemia, neuropathy, fatigue, nausea, and malnutrition, which significantly affect patients’ tolerance to the treatment. Some studies have shown that vitamin C could potentially augment chemotherapy’s tolerability, notably by boosting iron absorption, ameliorating anemia, and relieving pain and numbness in hands and feet. Nevertheless, the integration of vitamin C with chemotherapy to mitigate toxic side effects and enhance the quality of life for advanced pancreatic cancer patients has not been examined in any randomized controlled trials to date.

**Methods:**

A prospective, single-center, open-label, randomized controlled trial will be conducted at Fudan University Shanghai Cancer Center from September 2023 to September 2026. A total of at least 100 patients with advanced pancreatic adenocarcinoma exhibiting distant metastases will be recruited and randomly assigned to the chemotherapy group or the chemotherapy plus vitamin C group. The primary endpoint is the rate of anemia. Secondary endpoints include the rate of grade 3 neuropathy, change of numeric rating scale, quality of life, and overall survival.

**Discussion:**

This study aims to assess the impact of low-dose vitamin C on enhancing the quality of life for patients with metastatic pancreatic cancer undergoing gemcitabine and nab-paclitaxel chemotherapy.

**Trial registration:**

The trial was registered with the ClinicalTrials.gov (NCT06018883) on August 31, 2023.

## Administrative information

Note: the numbers in curly brackets in this protocol refer to SPIRIT checklist item numbers. The order of the items has been modified to group similar items (see http://www.equator-network.org/reporting-guidelines/spirit-2013-statement-defining-standard-protocol-items-for-clinical-trials/).

**Table:** 

Title {1}	Efficacy of Vitamin C on chemotherapy related anemia in pancreatic cancer: study protocol for a randomized controlled trial
Trial registration {2a and 2b}	clinicaltrial.gov (NCT06018883)
Protocol version {3}	1.2
Funding {4}	This study was jointly supported by National Natural Science Foundation of China (82172625) and Shanghai Charity Foundation (HYXH2021042).
Author details {5a}	Xinyue Wang ^a,b,c,d,1^, MD, Xinzhe Zhu ^a,b,c,d,1^, Yi Liu ^a,b,c,d,1^, MD,MD, He Liu ^a,b,c,d,1^, MD, Zhiwen Xiao ^a,b,c,d,1^, MD, PhD, Guopei Luo ^a,b,c,d,*^, MD, PhD ^a^Department of Pancreatic Surgery, Fudan University Shanghai Cancer Center, China. ^b^Department of Oncology, Shanghai Medical College, Fudan University, China. ^c^Shanghai Pancreatic Cancer Institute, China. ^d^Pancreatic Cancer Institute, Fudan University, China. ^*^Correspondence to: Guopei Luo (luoguopei@ fudanpci.org) No. 270, Dong’An Road, Xuhui District, Shanghai, 200032, China, Tel: 86–21-64175590–1308; Fax: 86–21-64,031,446.
Name and contact information for the trial sponsor {5b}	Fudan University Shanghai Cancer Center, No. 270, Dong’An Road, Xuhui District, Shanghai, 200,032, China, Tel: 021–64175590
Role of sponsor {5c}	The study was conducted independently, with the sponsor and funding bodies having no involvement in the design, data collection, management, analysis, interpretation, report writing, or the decision to submit for publication.

## Introduction

### Background and rationale {6a}

Pancreatic cancer is one of the most malignant tumors of the digestive system, often referred to as the “king of cancers” [1, 2]. Globally, pancreatic cancer ranks 12th in incidence and 7th in mortality among all malignant tumors. According to statistics from the American Cancer Society, it is anticipated that pancreatic cancer will rise to become the second leading cause of cancer-related mortality in the United States by 2030 [3]. Pancreatic cancer, a highly aggressive malignancy, has a 5-year survival rate below 10% [4]. Approximately 80% of cases are diagnosed at an advanced stage [5]. Chemotherapy, specifically the combination of gemcitabine and nab-paclitaxel, has been established as a primary treatment for advanced pancreatic cancer, as confirmed by the MPACT trial [6]. Nevertheless, this regimen’s associated side effects, such as anemia, neuropathy, fatigue, nausea, and malnutrition, have limited its tolerability [6, 7]. Compared to the FOLFIRINOX regimen, which combines fluorouracil, oxaliplatin, irinotecan, and calcium folinate, the nab-paclitaxel plus gemcitabine regimen shows a higher incidence of anemia [6, 7]. In a real-world study, the incidence of grade III or higher anemia reached 31.8% in patients with advanced pancreatic cancer treated with nab-paclitaxel in combination with gemcitabine [7].

Vitamin C, also known as ascorbate, is a vital nutrient for human health. It regulates metabolism, immune response, collagen production, and iron uptake. As an essential nutrient, the human body cannot synthesize vitamin C and must obtain it through dietary intake. Vitamin C deficiency is commonly found in patients with various malignant tumors, and the extent of deficiency correlates positively with the grade of the malignancy. In some patients with terminal stage malignancies, vitamin C can be completely absent. Furthermore, patients undergoing chemotherapy exhibit more pronounced vitamin C deficiency. Many cancer patients experience symptoms akin to scurvy during chemotherapy due to the significant reduction in vitamin C levels. This may be attributed to chemotherapy drugs interfering with the uptake and absorption of vitamin C [8]. Studies have shown that the concomitant use of vitamin C with chemotherapy does not affect the efficacy of the chemotherapeutic drugs. Instead, it has the potential to diminish toxic side effects related to chemotherapy and may even enhance the chemotherapy response in some cancer patients [9–11]. A clinical study conducted by Welsh et al. from the University of Iowa on patients with stage IV pancreatic cancer involved a single intravenous infusion of 50–125 g of vitamin C. The results indicated that plasma concentrations reached 20–25 mM 1 h post-infusion, without reaching the maximum dose, and no significant toxic side effects were observed [12]. Levine et al. from the National Institutes of Health studied 14 stage IV pancreatic cancer patients treated with gemcitabine and erlotinib treatment, supplemented with different doses of vitamin C (50, 75, 100 g). No vitamin C-related toxic side effects were observed during the trial, indicating the clinical safety of vitamin C [13]. Multiple studies have demonstrated that the combination of vitamin C can improve the tolerance of cancer patients to chemotherapy and radiotherapy, alleviate pain, and enhance patients’ compliance with chemotherapy [14, 15]. In 2011, a study showed that vitamin C could reduce the incidence of chemotherapy-induced vomiting, poor appetite, fatigue, depression, sleep disturbances, dizziness, and bleeding-related diseases in breast cancer patients, significantly improving their quality of life. More importantly, vitamin C can promote the absorption of iron supplements in the gastrointestinal tract, improving the anemic condition in cancer patients [16, 17]. For patients with iron-deficiency anemia, supplementing vitamin C combined with iron supplements resulted in over 85% of patients having a hemoglobin increase of more than 2 g/L [16]. However, randomized controlled trials investigating the combined use of vitamin C and chemotherapy to mitigate chemotherapy-induced adverse effects and enhance the quality of life in individuals with advanced pancreatic cancer are presently lacking.

This study aims to assess the impact of low-dose vitamin C on enhancing the quality of life for patients with metastatic pancreatic cancer undergoing gemcitabine and nab-paclitaxel chemotherapy. A total of 100 patients will be randomly allocated to either the experimental group (receiving gemcitabine and nab-paclitaxel, along with vitamin C) or the control group (receiving only gemcitabine and nab-paclitaxel). The evaluation parameters include the rate of anemia, incidence of hand/foot numbness, intensity of pain, quality of life, and overall survival, which will be measured at 4-week intervals.

### Objectives {7}

Primary objective:

The primary objective is to evaluate the efficacy of vitamin C on chemotherapy-related anemia in patients with advanced pancreatic cancer.

Secondary objective:

- To observe the treatment effect of vitamin C on chemotherapy-related hand/foot numbness in patients with advanced pancreatic cancer
- To observe the impact of vitamin C application on pain improvement in patients with advanced pancreatic cancer
- To observe the improvement in quality of life scores in patients with advanced pancreatic cancer following the application of vitamin C
- To evaluate the overall survival of participants from the date of randomization until the date of death due to any reason, whichever occurs first, evaluated for up to 24 months (time frame: at the end of cycle 1 with each cycle lasting 28 days)

### Trial design {8}

**Fig. 1:**
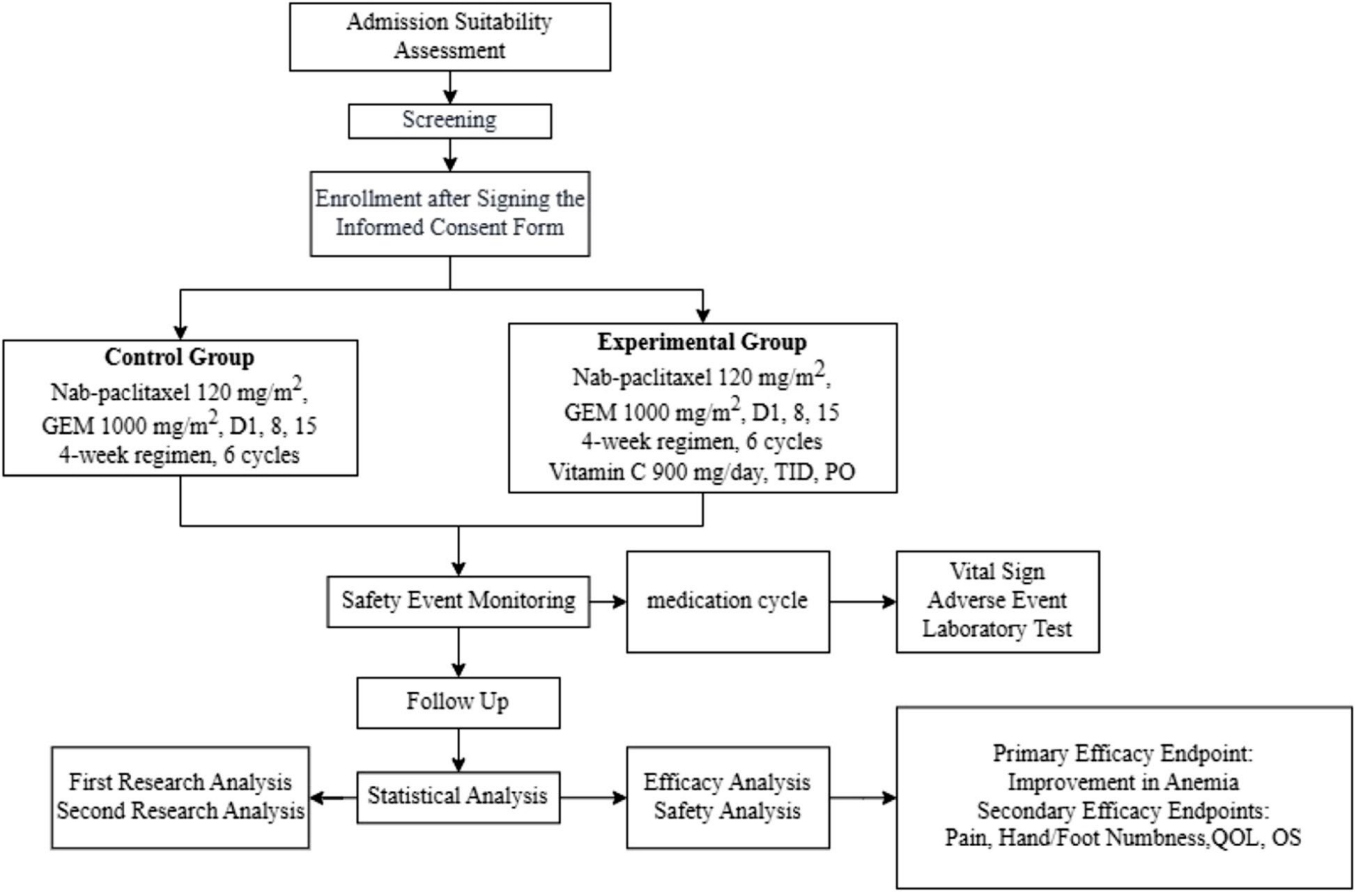
Trial flowchart

This study is a prospective, single-center, open-label, randomized controlled trial to examine whether combining vitamin C with chemotherapy is superior to chemotherapy alone in improving anemia, hand/foot numbness, pain, quality of life, and overall survival in patients with advanced pancreatic adenocarcinoma. Participants will be randomly assigned in a 1:1 ratio to either the experimental group (receiving gemcitabine and nab-paclitaxel, along with vitamin C) or the control group (receiving only gemcitabine and nab-paclitaxel). The flow chart of this study is displayed in Fig. 1.

## Methods: participants, interventions, and outcomes

### Study setting {9}

The research will take place at the Fudan University Shanghai Cancer Center, one of the premier cancer treatment facilities in China. The center handles over 2 million outpatient visits annually and provides care for 3000 to 4000 pancreatic cancer patients each year.

### Eligibility criteria {10}

Inclusion criteria:

- Ability to understand and the willingness to sign a written informed consent document
- Age ≥ 18 years and ≤ 80 years
- Eastern Cooperative Oncology Group (ECOG) performance status 0–2
- Histologically or cytologically confirmed metastatic pancreas adenocarcinoma
- Adequate organ performance based on laboratory blood tests
- Presence of at least of one measurable lesion in agreement to RECIST criteria
- Hemoglobin (Hgb) ≥ 8 g/dL
- The expected survival ≥ 3 months
- Women of childbearing potential and men must agree to use adequate contraception prior to study entry and for the duration of study participation

Exclusion criteria:

- Patients who have received any form of anti-tumor therapy
- The diagnosis was confirmed by pathology as non-adenocarcinoma of pancreas
- Inflammation of the digestive tract, including pancreatitis, cholecystitis, and cholangitis
- Pregnant or nursing women
- Glucose-6-phosphate dehydrogenase (G6PD) deficiency
- Severe and uncontrollable accompanying diseases that may affect protocol compliance or interfere with the interpretation of results, including active opportunistic infections or advanced (severe) infections, and diabetes that cannot be controlled after adequate clinical anti-hyperglycemia treatment according to guidelines, uncontrollable hypertension, cardiovascular disease (class III or IV heart failure as defined by the New York Heart Association classification, congestive heart failure (CHF), myocardial infarction in the past 6 months, unstable arrhythmia or unstable angina, cerebral infarction within 3 months, etc.)
- Renal insufficiency or dialysis
- History of allergic reactions attributed to compounds of similar chemical or biologic composition to gemcitabine, nab-paclitaxel, or other agents used in the study
- Other serious accompanying illnesses, which, in the researcher’s opinion, could seriously adversely affect the safety of the treatment
- Patients who are unwilling or unable to comply with study procedures

### Who will take informed consent? {26a}

In this clinical trial, the principal investigator or an authorized representative will be responsible for obtaining informed consent. The process involves a detailed explanation of the study’s background, benefits, and risks. Participants will be provided with a copy of the informed consent document, which contains details about the voluntary nature of participation, the confidentiality of personal data, and the right to withdraw at any moment without consequence. The informed consent process will be thoroughly documented in the participant’s medical records, and all forms are approved by the ethics committee before the commencement of the trial. The participant information materials and informed consent form are available from the corresponding author on request.

### Additional consent provisions for collection and use of participant data and biological specimens {26b}

The study does not involve the collection of additional data beyond the scope of the primary research objectives.

## Interventions

### Explanation for the choice of comparators {6b}

The comparator in this study is standard chemotherapy without vitamin C, as the primary objective is to determine the efficacy of vitamin C in treating chemotherapy-related anemia in advanced pancreatic adenocarcinoma with distant metastasis.

### Intervention description {11a}

A total of at least 100 participants with advanced pancreatic adenocarcinoma exhibiting distant metastases will be enrolled and randomly assigned in a 1:1 ratio. The control group receives albumin-bound paclitaxel at a dose of 120 mg/m^2^ plus gemcitabine at 1000 mg/m^2^. The albumin-bound paclitaxel/gemcitabine is administered on days 1, 8, and 15, with one cycle lasting 4 weeks and a rest period on the fourth week. The experimental group follows the same chemotherapy regimen as the control group, with the addition of vitamin C at a dose of 300 mg per administration, taken orally three times a day, daily. The study observes the effects of vitamin C in combination with albumin-bound paclitaxel and gemcitabine compared to chemotherapy alone on anemia, hand/foot numbness, pain, and quality of life in patients with advanced pancreatic adenocarcinoma with distant metastases. Safety events are assessed through relevant examinations before and after each treatment cycle, with radiological evaluations every two cycles during treatment, and every 3 months during the follow-up period to monitor disease progression.

### Criteria for discontinuing or modifying allocated interventions {11b}

Participants may discontinue the trial medication before the second study analysis. Reasons and dates of discontinuation will be recorded in both the original medical records and the case report form (CRF). Potential reasons for withdrawal or early termination include:

- The researcher’s judgment that continued trial medication may harm the participant’s health
- Clinical signs, laboratory results, and pregnancy indications consistent
- Receipt of concomitant treatment not allowed in the protocol, significantly affecting endpoint efficacy evaluation
- Significant comorbidities or other conditions affecting clinical status and study endpoints
- Poor participant compliance potentially interfering with endpoint efficacy analysis
- Participant or legal representative’s request to withdraw
- Loss to follow-up
- Death
- Specific requests from the organizing body

If the researcher decides to stop the trial medication due to overall health considerations, this does not necessarily mean termination of the trial for the participant. Follow-up visits will continue, and the participant’s data will still be included in the study analysis.

### Strategies to improve adherence to interventions {11c}

To ensure adherence to the interventions in this trial, participants are required to return to the clinic as scheduled for all medication cycles and related assessments. The number of medication cycles, the actual amount and duration of trial medication received per cycle, and reasons for deviating from the treatment plan will be recorded in both the original medical records and CRF to assess participant compliance. The clinical research coordinators (CRCs) and researchers will maintain close contact and communication with participants, clarifying any medical procedures and events during the study and informing them that low adherence may lead to their withdrawal from the trial. Monthly blood tests will be conducted to monitor the plasma concentration of vitamin C, ensuring participants’ daily intake of vitamin C and further enhancing adherence.

### Relevant concomitant care permitted or prohibited during the trial {11d}

Permitted concomitant medications and treatments:

- Routine standard supportive therapies are acceptable
- Treatment for any other underlying conditions, such as hypertension or hyperlipidemia, is allowed

Prohibited concomitant medications and treatments:

- Use of other clinical trial medications is not permitted during the study period
- Chemotherapeutic agents not specified in this protocol are prohibited

### Provisions for post-trial care {30}

After completion of the trial, participants will continue to receive standard care, with follow-ups conducted as needed.

### Outcomes {12}

Primary outcome:

The primary outcome will be the rate of anemia, referring to the proportion of participants who develop anemia following chemotherapy from the time of enrollment.

Secondary outcome:

- Rate of grade 3 neuropathy: Rate of grade 3 neuropathy after every cycle of chemotherapy;
- Change of numeric rating scale, NRS: Change of NRS and the administration of analgesic drugs after every cycle of chemotherapy. NRS is an 11-point numerical rating scale utilized for assessing the intensity of pain. It entails patients rating their pain on a scale from 0, representing no pain, to 10, representing the worst pain. Due to its sensitivity and ease of use, NRS is commonly employed in clinical practice for monitoring pain;
- Quality of life, QOL: Change of QOL after every cycle of chemotherapy. The quality of life of participants was assessed using the European Organization for Research and Treatment of Cancer Quality of Life Questionnaire Core 30 (EORTC QLQ-C30) version 3.0. The EORTC QLQ-C30 is a validated questionnaire developed to assess the quality of life in cancer patients [18]. It features nine multi-item scales, including five functional scales, three symptom scales, and a global health and quality-of-life scale, along with several single-item symptom measures;
- Overall survival, OS: OS of subjects from randomization to the time of death from any cause.

### Participant timeline {13}

Table 1 displays the timeline.

**Table 1:** Participant timeline

Project	Preliminary screening	Actual enrollment Treatment cycle 1			4-week regimen medication Treatment cycles 2–6			Follow-up
		Baseline screening	medication	Post-medication check	Pre-medication check	Medication	Post-medication check	
Imaging examination	X							
Informed consent form	X							
Pathological examination	X							
Qualification CT		X						
Eligibility criteria		X						
Actual grouping		X						
Demographic data		X						
Detailed medical history and comprehensive physical examination		X			X			
Laboratory examination of blood, urine, and stool samples, CRP		X		X	X		X	
Urine pregnancy test		X			X			
Quantitative CA19-9		X		X			X	
Other tumor markers		X		X			X	
Glucose-6-phosphate Dehydrogenase activity		X						
Electrocardiogram (ECG)		X			X			
ECOG performance status		X			X			
Hand-foot numbness assessment		X		X			X	
Pain score		X		X			X	
QOL score		X		X			X	
Abdominal and chest CT							X	
Control regimen administration			X			X		
Study regimen administration			X			X		
Adverse events		X	X	X	X	X	X	
Concomitant treatment		X	X	X	X	X	X	X
Efficacy evaluation								X

### Sample size {14}

Based on our center’s data, advanced pancreatic cancer patients undergoing two cycles of chemotherapy with albumin-bound paclitaxel and gemcitabine have a 69% incidence rate of anemia. We hypothesize that vitamin C supplementation could reduce this rate to 38%. To test this hypothesis, we plan a randomized control trial with a 1:1 allocation, targeting a sample size of 100 patients (50 per group), factoring in a 10% dropout rate. This size is calculated to achieve a power of 90% (1-β = 0.9) with a significance level of 0.05 (*α* = 0.05), allowing us to detect a significant difference in anemia incidence between the groups.

### Recruitment {15}

Participants will be recruited through posters and online platforms. Patient recruitment will be conducted at Fudan University Shanghai Cancer Center, and the recruitment period was from September 2023 to September 2024. We plan to recruit at least 100 participants, with a mean of 10 participants per month.

## Assignment of interventions: allocation

### Sequence generation {16a}

The randomization sequence will be generated with the sealed envelope method.

### Concealment mechanism {16b}

The use of opaque, sealed envelopes ensures the concealment of the allocation, preventing any potential tampering or identification of the allocation sequence before the envelopes are opened.

### Implementation {16c}

The list containing randomization numbers and the designated treatment groups is securely stored and accessible only to the designated independent staff member responsible for opening the sequentially numbered, opaque, sealed envelopes to allocate the assigned treatment to patients.

## Assignment of interventions: blinding

### Who will be blinded {17a}

Our study employs a single-blind design, wherein only the outcomes assessors are blinded to the treatment allocation to minimize the risk of bias in evaluating the results. Trial participants and care providers administering the treatments will be aware of the assigned treatment groups.

### Procedure for unblinding if needed {17b}

Since both care providers and participants are not blinded in this trial, the procedure for unblinding is not applicable.

## Data collection and management

### Plans for assessment and collection of outcomes {18a}

#### Screening phase

Researchers obtain informed consent from participants who meet the preliminary screening criteria. Only after the participants have signed the latest version of the informed consent form can the next step of screening proceed.

Following this, researchers will evaluate the tumor characteristics of participants using imaging data in line with the inclusion/exclusion criteria of the protocol. This includes abdominal CT/MRI scans (plain and enhanced), endoscopic ultrasonography (EUS) as a supplementary method to CT, and PET/CT scans not as a routine method. The evaluation will cover aspects such as tumor location (head, body, or tail of the pancreas), number, size, extent of invasion into surrounding pancreatic tissues, and distant metastasis. These evaluations must be documented in the patient’s medical records. Additionally, the imaging data will be collected and preserved as source documents.

#### Baseline assessment

Before enrollment, it is essential to ensure participants meet the inclusion/exclusion criteria and to gather baseline data in the following areas:

- Abdominopelvic CT scans (plain and enhanced)
- If participants exhibit clinical symptoms or signs suggestive of distant tumor metastasis, additional imaging studies and/or laboratory tests may be conducted:
- MRI for suspected brain and nervous system metastases
- ECT for suspected bone metastases
- Fine-needle aspiration cytology for suspected superficial lymph node metastases
- Routine blood, urine, and stool tests, including occult blood test, and electrocardiogram (ECG)
- Liver function tests (AST, ALT, GGT, total bilirubin, albumin, prealbumin), renal function (creatinine), electrolytes, blood glucose, serum amylase, and glycated hemoglobin
- Coagulation function (prothrombin time)
- Quantitative CA19-9 (requiring precise figures within 0–6000 U/mL) and other tumor biomarkers
- Urine pregnancy test for women of childbearing age
- G6PD activity test, if needed
- Vital signs, height, weight, and physical examination
- QOL scoring for cancer patients. The quality of life of participants was evaluated using the European Organization for Research and Treatment of Cancer Quality of Life Questionnaire Core 30 (EORTC QLQ-C30) version 3.0. This questionnaire, designed specifically for cancer patients, consists of nine multi-item scales: five functional scales, three symptom scales, and a global health and quality-of-life scale, along with several single-item symptom measures. It has been demonstrated to be a reliable and valid instrument for assessing the quality of life in cancer patients in diverse clinical research settings [18]
- Collection of participants’ medical history including diabetes, pancreatitis, benign pancreatic tumors, treatment history, and allergies, to be recorded in the medical records

Researchers will determine the staging of pancreatic cancer in participants based on imaging reports, other laboratory tests, and clinical signs and symptoms, which will be documented in the medical records.

### Intervention phase

#### Pre-medication examination

The following procedures must be completed within 7 days prior to medication administration:

- Abdominal and pelvic CT scan, both plain and enhanced
- Routine blood, urine, and stool tests, including occult blood test, and electrocardiogram (ECG)
- Liver function tests (including AST, ALT, γ-GT, total bilirubin, albumin, and prealbumin), renal function test (creatinine), electrolytes, blood glucose, serum amylase, and glycated hemoglobin
- Coagulation function, specifically prothrombin time
- Quantitative CA19-9 (requiring precise numbers within the range of 0–6000 U/mL) and other tumor markers
- Hepatitis B panel and anti-HCV antibody test (if not previously completed)
- Urinary pregnancy test (for women of childbearing age)
- Vital signs, height, weight, and physical examination
- QOL scoring
- ECOG performance status
- Pain assessment
- Anemia evaluation
- Collection of the patient’s history of diabetes, pancreatitis, benign pancreatic tumors, previous malignancies, treatment history, and allergies, to be recorded in the medical record

#### During medication

In cases of adverse events caused by medication during the treatment period, researchers are responsible for implementing the necessary monitoring and treatment and must accurately record these events in the patient’s medical records.

#### Post-medication examination

The following relevant examination must be completed within one day after the conclusion of the medication study:

- Hemoglobin levels
- Hand and foot numbness scoring
- Pain scoring: NRS
- QOL scoring

#### Follow-up phase

After subjects have undergone treatment with the study medication, researchers are required to conduct follow-up with the subjects through face-to-face visits to calculate and analyze the 1-year, 2-year, and 5-year survival rates.

### Plans to promote participant retention and complete follow-up {18b}

To promote participant retention and complete follow-up, our study will provide detailed explanations of the condition and treatment plan during out-patient interviews, and ensure regular follow-up with each participant to monitor their health status and address any concerns.

### Data management {19}

#### Data processing

Researchers are required to enter the data of enrolled subjects into the CRF. Patients who fail screening are not required to be documented in the CRF. Researchers must ensure that the data entered in the CRF is accurate and complete, and original records are also to be preserved in their entirety. The CRF for each enrolled subject must be completed in a timely manner. Once reviewed, the completed CRFs are to be handed over to the data manager of this trial for data entry and management.

#### Data entry

The data entry and management are the responsibility of the designated data management unit. Data administrators use computer software to develop data entry programs for the purpose of entering and managing data. To ensure the accuracy of the data, it should be proofread by another person after completion of the entry process.

#### Medical information coding

Medical information coding will utilize the following tools:

- MedDRA 11.0 (for medical history and adverse events)
- WHO Drug 2008.03 (for concomitant medications)
- NCI-CTCAE 3.0 (for toxic reactions)

#### Data verification

The database that has been established will be verified by the principal investigator, organizers, data management personnel, and statistical analysis staff. The database will be locked after confirming the research dataset and the statistical analysis plan.

#### Record retention

In accordance with Good Clinical Practice (GCP) guidelines, the principal investigator must retain all documents related to the clinical trial, including patient medical records, medication dispensation records, signed informed consent forms, ethical committee approvals, and other relevant materials, for a period of 5 years following the conclusion of the clinical trial.

#### Authority over original data/documents

In clinical trials, any observations and examination results should be recorded by the researchers promptly, accurately, completely, in a standardized manner, and truthfully in the medical records and correctly transcribed into the CRF. Changes should not be made arbitrarily. In case of errors in recording, any corrections made by the researchers should maintain the clarity and recognizability of the original records, with the corrections being signed and dated by the person making them. The original data/documents must be properly preserved at the research center in accordance with ICH-GCP (International Committee on Harmonization of Good Clinical Practice) guidelines and local regulatory requirements.

Higher-level supervisory departments and drug regulatory authorities have the authority to monitor, audit, and inspect the implementation process of the clinical trials and the original data/source documents. Monitors, auditors, and regulatory authorities are entitled to review all trial-related original data/source documents but do not have the authority to make modifications. If any issues are identified, they must inform the researchers, who should then make the necessary corrections and sign off on them.

#### Confidentiality {27}

Research reports are strictly prohibited from identifying subjects by their names. These reports are solely intended for research purposes. Utmost efforts will be exerted to maintain the confidentiality of subjects’ personal medical data.

#### Plans for collection, laboratory evaluation, and storage of biological specimens for genetic or molecular analysis in this trial/future use {33}

Tissue and blood samples were collected for molecular testing and future analysis.

## Statistical methods

### Statistical methods for primary and secondary outcomes {20a}

#### Primary endpoint

Rate of anemia: This is defined as the proportion of subjects who develop anemia after chemotherapy from the time of enrollment. The difference in rate of anemia between the experimental group and the control group will be compared using the chi-square test. A stratified analysis will be conducted for patients with late-stage tumors post-surgery and those with late-stage tumors without surgery.

#### Secondary endpoints

- Rate of grade 3 neuropathy: This refers to the occurrence of grade 3 neuropathy in subjects after chemotherapy from the time of enrollment. The difference in incidence rates between the experimental and control groups will be analyzed using the chi-square test.
- NRS: This involves the assessment of changes in pain scores and the use of analgesic medication after chemotherapy from the time of enrollment. An increase in pain scores, additional dosage of analgesics, or the combined use of other pain relief medication will be considered as an exacerbation of pain. The difference in the rate of pain exacerbation between the experimental and control groups will be compared using the chi-square test.
- QOL in cancer patients: It will be evaluated at each assessment and compared to the baseline. A descriptive analysis will be conducted using the analysis of covariance (ANCOVA) model, with the baseline as a covariate. The effect of each assessor will be considered as a random effect in the model.

### Interim analyses {21b}

There is no interim analysis plan for this trial.

### Methods for additional analyses (e.g., subgroup analyses) {20b}

Subgroup analysis was not planned for this trial.

### Methods in analysis to handle protocol non-adherence and any statistical methods to handle missing data {20c}

Through a comprehensive sensitivity analysis that examines the impact of various missing data handling methods (such as multiple imputation, Bayesian methods) on key outcomes, we will employ appropriate statistical techniques to ensure the accuracy and robustness of our trial results.

### Plans to give access to the full protocol, participant-level data, and statistical code {31c}

The datasets examined during the current study, along with the statistical code and the complete protocol, can be obtained from the corresponding author upon a reasonable request. Interested parties can obtain the datasets, statistical code, and the full study protocol used in this research by submitting a reasonable request to the corresponding author.

## Oversight and monitoring

### Composition of the coordinating center and trial steering committee {5d}

This clinical trial is organized by the Fudan University Shanghai Cancer Center, with the Pancreatic Tumor Research Institute, Department of Pancreatic Surgery at Fudan University, acting as the coordinating center. Researchers and assistants are required to be fully versed in the study’s protocol, treatments, and relevant duties. They must maintain a list of delegated personnel involved in significant research activities and are responsible for the records of all consented and screened participants. In cases where patients do not meet screening criteria, reasons must be documented in their source files. During monitoring visits, researchers or authorized individuals are to review data, address queries, and permit direct access to patient records for data verification, ensuring timely and accurate completion of the CRF.

The Institution Review Board of Shanghai Cancer Center serves as the steering committee, tasked with supervising the trial’s overall execution, ensuring compliance with ethical standards, and monitoring the progress of the study.

The Radiology Department of Fudan University Shanghai Cancer Center serves as the third-party imaging review center, while the Pathology Department of the same institution functions as the third-party pathology review center. Additionally, the Biostatistics Unit operates under the auspices of the Clinical Statistics Center at Fudan University Shanghai Cancer Center.

### Composition of the data monitoring committee, its role and reporting structure {21a}

An independent data monitoring committee will oversee participant safety and data integrity.

Based on periodic evaluations of accumulating data, the committee will advise the steering committee and sponsor on whether to continue the study without modifications, implement modifications, or terminate the study.

### Adverse event reporting and harms {22}

Researchers will closely monitor adverse events (AEs) in participants throughout the clinical study. This monitoring extends from the time of enrollment until the end of the study or early withdrawal of the participant. All reported AEs by the subjects or observed by medical personnel must be documented in both the medical records and the CRF. This documentation should minimally include the name of the AE, the time of its occurrence and resolution, its severity, whether corrective treatment was administered, the outcome of the AE, and its relationship to the trial medication.

For serious adverse events (SAEs), it is required to report them within 24 h of becoming aware. In the event of an SAE during the clinical research, the researcher must immediately administer appropriate treatment to the participant and report the incident to the ethics committee of the clinical trial unit via phone or fax within 24 h. A written report should also be submitted to the same authorities. The occurred SAE must be fully recorded in both the CRF and the SAE report form. Additionally, all SAEs that occur within 30 days after the last administration of the trial drug or upon the termination of the trial should be duly recorded and reported. Upon receiving new information, it should be reported within 48 h. For all SAEs, the researcher is required to follow-up until the event is resolved, returns to baseline level, is confirmed as unresolved/permanent, the participant switches to other antitumor treatments, or in case of death. Medical documentation related to the SAEs should be recorded in both the original documents and the CRF, including laboratory and ancillary test results. The researcher is responsible for analyzing and determining the causal relationship between the SAEs and the trial medication from a clinical perspective. The final report must be submitted within 48 h of obtaining the final information. If an SAE remains unresolved 30 days after the conclusion of the trial (not yet returned to baseline level, not considered as a permanent disability, and/or no death has occurred), the final report should be submitted in accordance with the reporting procedures of the ethics committee and the organizing body.

### Frequency and plans for auditing trial conduct {23}

The Expert Committee plays a pivotal role in clinical trials, participating in discussions on the trial protocol and providing guidance on scientific and ethical aspects of its design. Throughout the trial, this committee supervises and manages the quality of the study, urging resolution of quality issues and participating in the review of the final trial report to ensure that the research conclusions are accurate and consistent with the trial data.

During the clinical research process, supervisory authorities regularly conduct inspections to ensure compliance with the research protocol and Good Clinical Practice (GCP). Inspectors review original documents and check the completeness of CRF. Researchers and research sites must allow inspectors and relevant regulatory bodies access to original documents during these audits.

Research centers are also subject to inspections by drug regulatory authorities. These inspections involve reviewing trial records, documentation, and relevant regulatory files. During these inspection visits and data audits, researchers and associated personnel must be present and have sufficient time to participate. At all times, the privacy of participants is paramount and must be protected and respected. Typically, researchers are notified prior to these visits.

Government departments periodically inspect the implementation of trials to ensure that the execution of the protocol complies with legal and ethical standards.

### Plans for communicating important protocol amendments to relevant parties (e.g., trial participants, ethical committees) {25}

If necessary, during the research process, the study protocol can be revised to guide the next steps of the research. Any protocol amendments must adhere to GCP regulatory requirements. The modifications to the protocol are drafted by the researchers and organizers based on the progress of the study and must be reviewed and approved by the ethics committee before implementation. The approved amendments by the ethics committee must become an integral part of the revised protocol.

### Dissemination plans {31a}

The results of this study may be published in medical journals or magazines or used for teaching purposes. Additionally, in accordance with the requirements of local health agencies, this study and its results may be submitted for inclusion in all relevant health research institution registries and published on health institution research registry websites (such as ClinicalTrials.gov). The selection of the first author is determined by various factors, including but not limited to participation in the study, contributions to the development of the protocol, and involvement in the analysis and input in the study manuscript, related abstracts, and presentations.

## Discussion

In the management of advanced pancreatic cancer, chemotherapy is a cornerstone treatment. Its efficacy, however, is often overshadowed by side effects including anemia, numbness in hands and feet, fatigue, nausea, and malnutrition, which significantly challenge patient tolerance [6, 7]. Emerging studies have indicated that vitamin C may enhance chemotherapy tolerability by improving iron absorption, reducing anemia, and alleviating neuropathic pain and numbness [14, 15]. Despite these potential benefits, the role of vitamin C in conjunction with chemotherapy to mitigate its toxic side effects and improve quality of life in patients with advanced pancreatic cancer has not been thoroughly investigated in randomized controlled trials.

Given the potential of vitamin C to improve chemotherapy tolerability and enhance patients’ quality of life, our proposed clinical study focuses on treating chemotherapy-related anemia in advanced pancreatic cancer with vitamin C. This trial is designed to evaluate the efficacy of vitamin C in alleviating anemia, neuropathic symptoms like hand and foot numbness, and pain, thereby potentially enhancing the overall quality of life for these patients. The insights gained from this study could be pivotal in guiding the use of vitamin C in oncological treatment regimes, aiming to boost patient compliance and tolerance towards chemotherapy and radiotherapy, ultimately seeking to improve therapeutic outcomes in pancreatic cancer treatment.

## Trial status

As of submitting this manuscript, the study is currently in the stage for participant recruitment. We are working with protocol version 1.2, finalized on August 1, 2023. The commencement of recruitment is September 2023, with the anticipation of completing this phase by September 2024.

## Data Availability

The datasets utilized and examined in this study can be obtained from the principal investigator (Guopei Luo) upon reasonable request.

## Abbreviations

ECOG: Eastern Cooperative Oncology Group
Hgb: Hemoglobin
G6PD: Glucose-6-phosphate dehydrogenase
CHF: Congestive heart failure
CRF: Case report form
CRCs: Clinical research coordinators
NRS: Numeric rating scale
QOL: Quality of life
OS: Overall survival
EUS: Endoscopic ultrasonography
PET: Positron emission tomography
CT: Computed tomography
MRI: Magnetic resonance imaging
ECT: Emission computed tomography
ECG: Electrocardiogram
AST: Aspartate aminotransferase
ALT: Alanine aminotransferase
GGT: Gamma-glutamyl transferase
CA19-9: Carbohydrate antigen 19–9
HCV: Hepatitis C virus
GCP: Good Clinical Practice
ICH-GCP: International Committee on Harmonization of Good Clinical Practice
AEs: Adverse events
SAEs: Serious adverse events

## References

[1] Chen W, Zheng R, Baade PD, Zhang S, Zeng H, Bray F, et al. Cancer statistics in China, 2015. CA Cancer J Clin. 2016;66:115–32. 26808342 10.3322/caac.21338

[2] Siegel RL, Miller KD, Jemal A. Cancer statistics, 2017. CA Cancer J Clin. 2017;67:7–30. 28055103 10.3322/caac.21387

[3] Mizrahi JD, Surana R, Valle JW, Shroff RT. Pancreatic cancer. Lancet Lond Engl. 2020;395:2008–20. 10.1016/S0140-6736(20)30974-0 32593337

[4] Siegel RL, Miller KD, Fuchs HE, Jemal A. Cancer statistics, 2021. CA Cancer J Clin. 2021;71:7–33. 33433946 10.3322/caac.21654

[5] Ryan DP, Hong TS, Bardeesy N. Pancreatic adenocarcinoma. N Engl J Med. 2014;371:1039–49. 25207767 10.1056/NEJMra1404198

[6] Von Hoff DD, Ervin T, Arena FP, Chiorean EG, Infante J, Moore M, et al. Increased survival in pancreatic cancer with nab-paclitaxel plus gemcitabine. N Engl J Med. 2013;369:1691–703. 24131140 10.1056/NEJMoa1304369 PMC4631139

[7] Chun JW, Lee SH, Kim JS, Park N, Huh G, Cho IR, et al. Comparison between FOLFIRINOX and gemcitabine plus nab-paclitaxel including sequential treatment for metastatic pancreatic cancer: a propensity score matching approach. BMC Cancer. 2021;21:537. 33975561 10.1186/s12885-021-08277-7 PMC8114681

[8] Mamede AC, Tavares SD, Abrantes AM, Trindade J, Maia JM, Botelho MF. The role of vitamins in cancer: a review. Nutr Cancer. 2011;63:479–94. 21541902 10.1080/01635581.2011.539315

[9] Espey MG, Chen P, Chalmers B, Drisko J, Sun AY, Levine M, et al. Pharmacologic ascorbate synergizes with gemcitabine in preclinical models of pancreatic cancer. Free Radic Biol Med. 2011;50:1610–9. 21402145 10.1016/j.freeradbiomed.2011.03.007 PMC3482496

[10] Frömberg A, Gutsch D, Schulze D, Vollbracht C, Weiss G, Czubayko F, et al. Ascorbate exerts anti-proliferative effects through cell cycle inhibition and sensitizes tumor cells towards cytostatic drugs. Cancer Chemother Pharmacol. 2011;67:1157–66. 20694726 10.1007/s00280-010-1418-6 PMC3082037

[11] Fujita K, Shinpo K, Yamada K, et al. Reduction of adriamycin toxicity by ascorbate in mice and guinea pigs. Cancer Res. 1982;42(1):309–16. 7053858

[12] Welsh JL, Wagner BA, van’t Erve TJ, Zehr PS, Berg DJ, Halfdanarson TR, et al. Pharmacological ascorbate with gemcitabine for the control of metastatic and node-positive pancreatic cancer (PACMAN): results from a phase I clinical trial. Cancer Chemother Pharmacol. 2013;71:765–75. 23381814 10.1007/s00280-013-2070-8 PMC3587047

[13] Monti DA, Mitchell E, Bazzan AJ, Littman S, Zabrecky G, Yeo CJ, et al. Phase I evaluation of intravenous ascorbic acid in combination with gemcitabine and erlotinib in patients with metastatic pancreatic cancer. PLoS ONE. 2012;7:e29794. 22272248 10.1371/journal.pone.0029794 PMC3260161

[14] Vollbracht C, Schneider B, Leendert V, Weiss G, Auerbach L, Beuth J. Intravenous vitamin C administration improves quality of life in breast cancer patients during chemo-/radiotherapy and aftercare: results of a retrospective, multicentre, epidemiological cohort study in Germany. In Vivo. 2011;25(6):983–90. 22021693

[15] Abiri B, Vafa M. Vitamin C and Cancer: The role of vitamin C in disease progression and quality of life in cancer patients. Nutr Cancer. 2021;73:1282–92. 32691657 10.1080/01635581.2020.1795692

[16] Sourabh S, Bhatia P, Jain R. Favourable improvement in haematological parameters in response to oral iron and vitamin C combination in children with iron refractory iron deficiency anemia (IRIDA) phenotype. Blood Cells Mol Dis. 2019;75:26–9. 30594846 10.1016/j.bcmd.2018.12.002

[17] Gilreath JA, Rodgers GM. How I treat cancer-associated anemia. Blood. 2020;136:801–13. 32556170 10.1182/blood.2019004017

[18] Aaronson NK, Ahmedzai S, Bergman B, Bullinger M, Cull A, Duez NJ, et al. The European Organization for Research and Treatment of Cancer QLQ-C30: a quality-of-life instrument for use in international clinical trials in oncology. J Natl Cancer Inst. 1993;85:365–76. 8433390 10.1093/jnci/85.5.365

